# Self-Regulation Shift Theory: A Dynamic Personal Agency Approach to Recovery Capital and Methodological Suggestions

**DOI:** 10.3389/fpsyg.2018.01738

**Published:** 2018-09-21

**Authors:** Charles C. Benight, Aaron Harwell, Kotaro Shoji

**Affiliations:** ^1^Department of Psychology, University of Colorado, Colorado Springs, Colorado Springs, CO, United States; ^2^Trauma, Health, and Hazards Center, University of Colorado, Colorado Springs, Colorado Springs, CO, United States

**Keywords:** post-trauma adaptation, social cognitive theory, self-regulation shift theory, coping self-efficacy, self-regulation

## Abstract

Recovery capital highlights person and environmental resources associated with overcoming significant life challenges. This paper utilizes social cognitive theory as a framework for understanding how recovery capital functions in trauma adaptation. This theory outlines the bidirectional, dynamic interactions among person (e.g., cognitive and affective), behavioral (e.g., problem focused coping), and environmental variables (i.e., triadic reciprocal determinism). The value of this approach to understanding human adaptation to trauma is that it targets the self-regulatory processes that unfold for trauma survivors as they attempt to put their lives back together. Self-regulation shift theory (SRST), as an extension to social cognitive theory, is offered to explain how self-regulation is involved in both positive and negative adjustment. The theory uses a dynamical systems approach and highlights the mechanisms related to non-linear shifts in both positive and negative trauma recovery. According to SRST, trauma recovery may not be linear with threshold shifts (i.e., bifurcations) from one organized state (broken self) to another (empowered self). Coping self-efficacy perceptions are a critical factor influencing these threshold shifts. This paper concludes with a brief review of study designs and analytic procedures that can facilitate the application of non-linear dynamic research in this area.

## Self-Regulation Shift Theory: a Personal Agency Approach to Recovery Capital

Recovery capital encompasses personal attributes, environmental resources, and social connections (Cloud and Granfield, 2008). Cloud and Granfield (2008) applied this concept to management of substance misuse. One could argue that resiliency following trauma also requires extensive use of personal, environmental, and social resources (i.e., recovery capital) (Hobfoll, 1991). Resilience is a broad term that has been defined in a number of ways. For the purposes of this paper we are adopting Luthar et al. (2000) definition: “resilience refers to a dynamic process encompassing positive adaptation within the context of significant adversity” (p. 543). This dynamic process encompasses a myriad of positive adaptive outcomes following trauma (e.g., posttraumatic growth, rebounding from distress, and positive meaning making). We are not advocating that all positive outcomes are the same. Indeed, it is possible that the dynamic utilization of recovery capital may promote differential positive adaptation (e.g., reduction in symptoms versus enhanced meaning in life). A theoretically based approach is critical to help understand how recovery capital operates in promoting resiliency following trauma. Social cognitive theory offers a useful and empirically supported framework.

Benight and Bandura (2004) utilized social cognitive theory (SCT) to explain the dynamic interplay among these resources along with coping behavior to explain the cognitive, affective, and ultimate motivational processes involved with human adaptation following trauma. They suggested traumatic stress adaptation can best be understood by describing the bidirectional, dynamic interactions among person (e.g., cognitive and affective), behavioral (e.g., coping), and social contextual variables (e.g., social support). Bandura (1991) refers to the bidirectional influence as triadic reciprocal determinism suggesting that each component can have a deterministic influence over the other resource. In essence, SCT offers a dynamic framework for how recovery capital influences trauma recovery as it unfolds across time. Central to this interactive process is the human capability for self-regulation. Bandura (1991) described self-regulation as encompassing individuals’ capacity for monitoring their performance (self-observation), evaluating their behavior against standards (judgment), and their affective response to this evaluation (self-reaction). In this paper, we will explore self-regulation shift theory (SRST), an extension of SCT that highlights the self-regulatory process that is central to positive adaptation following trauma. Before introducing this new theoretical model it is important to review the primary trauma adaptation theories to put the new approach into context.

There are several theoretical approaches describing adaptation after trauma that integrate personal and social resources. Beyond SCT which was just briefly described, anxiety buffer disruption theory (ABDT) (Pyszczynski and Kesebir, 2011), conservation of resources theory (Hobfoll, 1991), social support theory (Haber et al., 2007), and attachment theory (Bowlby, 1969), all offer frameworks for understanding the adaptational process following trauma.

These relatively broad theories all emphasize, to a greater or lesser degree, the importance of recovery capital (e.g., personal, social, and environmental resources). SCT highlights the importance of the personal resources in relationship to social context with particular emphasis on the belief in individual agency in dealing with often uncontrollable posttraumatic environmental demands (Benight and Bandura, 2004). Conservation of resources theory (COR) proposes that individuals seek to gain and retain valued resources that include individual, material, and social resources (Hobfoll, 1991). Anxiety buffer disruption theory highlights the existential buffer of one’s cultural world view of a benevolent world that is shattered from the trauma (Pyszczynski and Kesebir, 2011). Social support theory (SST) and attachment theory (AT), when applied to trauma, focus more on the importance of the social relationships and connectedness as paramount in trauma adaptation (Bowlby, 1969; Haber et al., 2007). Although it is beyond the scope of this paper to review the substantive literature on all of these theories, it is important to note that there is support for each of these theories in predicting posttraumatic outcomes (SCT; see Benight and Bandura, 2004 and Luszczynska et al., 2009 for reviews; Sumer et al., 2005; Boehmer et al., 2007; Bosmans et al., 2013, 2015; Singer et al., 2016; Chung et al., 2017; COR; see Hobfoll, 2001 for review; Freedy et al., 1992, 1994; Kaiser et al., 1996; Norris et al., 1999; Smith and Freedy, 2000; Sattler et al., 2002, 2006; Hobfoll et al., 2009; Lambert et al., 2018; ABDT; Abdollahi et al., 2011; Edmondson et al., 2011; Chatard et al., 2012; Vail et al., 2018; SST; see Cohen, 1988; Kaniasty, 2005; Uchino, 2006 for reviews; Robinaugh et al., 2011; Kaniasty, 2012; AT; Solomon et al., 2008; Alexander, 2009; Besser and Neria, 2010).

Clearly there is abundant research that supports the different theoretical models listed above. All of these theories include a dynamic change element related to adaptation across time (e.g., triadic reciprocal determinism, SCT; loss and gain spirals, COR; anxiety buffer disruption, ABDT; social support deterioration deterrence, SST; relational transactions, AT). In reviewing the literature related to these theories the methodology utilized to understand these changes across time have either ignored the time component using cross-sectional designs, or exclusively relied on longitudinal linear modeling techniques (e.g., path analyses, cross-lagged panel designs). We did not identify any studies, beyond SCT, that have evaluated the change processes involved in a non-linear way. This highlights the dominant assumption in this work that trauma adaptation evolves across time in a linear fashion. We argue below that SCT offers the most comprehensive dynamic theoretical framework with extensive empirical support that captures the elements of recovery capital. Self-regulation is fundamental to healthy adaptation and is central to SCT. We introduce in this paper a new theoretical model that targets possible non-linear trajectories with an emphasis on the self-regulation process outlined in SCT.

### Social Cognitive Theory and Trauma Adaptation

Trauma can be conceptualized as a massive, sudden, loss of critical personal, and social resources (Hobfoll, 1991). Such loss challenges existing self-regulatory capabilities often due to the suddenness and novelty of the experience (Benight and Bandura, 2004). Self-regulation is the management of valued personal goals (e.g., feeling normal again after a physical assault) through the ability to self-reflect and project probability of future goal attainment. This unique human attribute sets the stage for on-going coping adjustments as environmental and behavioral information is evaluated in a systemic feedback process (i.e., self-regulation). Positive adjustment is attained as self-regulation attempts gain traction with improvements in environmental conditions through effective coping. For example, a woman challenged with recovery from a sexual assault must manage both internal and external environmental hurdles such as possible intrusions of the attack and hyperarousal, while concurrently navigating the often challenging legal system. Through effective utilization of social, physical, and personal resources (i.e., recovery capital) she is able to manage her inner distress and navigate her legal challenges leading to an increasing sense of coping capability. Indeed, the self-evaluation of effective coping is a crucial personal resource and is often referred to as self-efficacy. We argue that coping self-efficacy (CSE) following trauma is central to ultimate empowerment, growth, and healthy recovery.

Self-efficacy is a critical self-appraisal personal resource that guides coping processes through self-evaluation. Indeed, these appraisals form a central self-belief about being able to handle both internal (e.g., intrusive thoughts) and external demands (social constraints). CSE is predictive of coping across multiple types of trauma (for reviews see Benight and Bandura, 2004; Luszczynska et al., 2009).

The value of a construct is based on its predictive power (i.e., amount of variance it explains). The effect for CSE as a predictor in longitudinal studies on mass trauma for posttraumatic outcomes (e.g., posttraumatic distress, substance use, and positive affect) is stronger than other predictors often evaluated in post-traumatic recovery studies. The effect sizes for CSE *r’*s ranging from -0.55 to -0.62 (Luszczynska et al., 2009) compared to *r’*s ranging from ±0.17 to 0.35 (e.g., dissociation, social support, and previous psychopathology) (Ozer et al., 2008).

If the self-regulatory process is central to trauma adaptation and CSE appraisals to manage this unfolding process are crucial, it follows then that CSE judgments should be predictive of outcomes across a multitude of trauma recovery contexts. The science has borne this out.

Coping self-efficacy was a significant predictor of post-trauma recovery outcomes following a wide variety of trauma experiences. These include childhood trauma (Cieslak et al., 2008; Singer et al., 2016), domestic violence (Benight et al., 2004; Lambert et al., 2013), war (Solomon et al., 1991; Smith et al., 2013; Chung et al., 2017), hurricanes (Benight et al., 1999; Hirschel and Schulenberg, 2009; Wadsworth et al., 2009), and terrorist attacks (Benight et al., 2000). Further, longitudinal research on disaster survivors (Benight and Harper, 2002; Bosmans et al., 2013), victims of acute physical injuries (Flatten et al., 2008; Bosmans et al., 2015), and survivors of motor vehicle accidents (Benight et al., 2008) indicated that CSE is a key mechanism through which initial distress influences subsequent post-traumatic symptoms. Most recently a general sense of mastery, which is another term for general self-efficacy, was found to moderate the effect of trauma on all-cause mortality (Elliot et al., 2018). Collectively, these studies strongly suggest that an individual’s sense of agency (i.e., degree of CSE) plays a pivotal role in trauma recovery. Although most of the aforementioned studies focused on negative outcomes following trauma, CSE also predicts positive adaptation.

### Positive Posttraumatic Adaptation and CSE

Research on CSE and positive outcomes following trauma have focused on posttraumatic growth (PTG; Cieslak et al., 2009), making meaning (Blackburn and Owens, 2015), positive affect (Luszczynska et al., 2009), and resilience (deRoon-Cassini et al., 2010). Cieslak et al. (2009), looked at posttraumatic growth (e.g., new appreciation for life, personal empowerment, and spiritual changes) following hurricane Katrina in individuals coping with HIV. They found CSE perceptions were significantly predictive of PTG in individuals with higher levels of posttraumatic distress. This suggests an important role for self-appraisals of capability in development of PTG. Blackburn and Owens (2015) found a significant relationship between self-efficacy beliefs and presence of meaning (i.e., values and feeling of purpose in life) in combat veterans (*r* = 0.63, *p* < 0.01). Luszczynska et al. (2009), in their meta-analytic review, found CSE was positively correlated with positive affect (e.g., joy, love, interest, or pride) in two mass trauma studies. deRoon-Cassini et al. (2010) studied the trajectories of distress levels in a sample of hospitalized trauma survivors. Lacks of symptoms were utilized to characterize the “resilient” group. CSE was an important predictor of posttraumatic trajectories with higher acute CSE predicting greater odds of membership in the resilience group versus the chronic trajectory. However, importantly, this effect was qualified by the finding that early high CSE was associated with the delayed PTSD onset group, demonstrating the complexity of adaptation over time. Collectively, the literature on CSE and positive outcomes following trauma has generally shown a strong positive relationship.

The studies on CSE as a predictor of outcomes following trauma, although strongly predictive, have all utilized a linear, somewhat simplistic approach, to explain a very complex dynamic process. Indeed, Cloud and Granfield (2008) suggested that recovery capital is based on the utilization of important environmental, social, and personal resources as an individual attempts to initiate and maintain healthy adaptation over time. This underscores the need for a more refined theoretical and empirical approach to understand how these interactions unfold. The deRoon-Cassini et al. (2010) findings that on one hand CSE perceptions were predictive of membership in the resilient group and also may be linked to a delayed distress group support this point. This complexity in how adaptation evolves across time is central to our argument that recovery resources in trauma adaptation must be viewed in a dynamic way. Indeed, the underlying dynamic process of traumatic stress adaptation remains largely unknown. SRST (Benight et al., 2017) provides a novel approach to understand this dynamic evolving process.

### Self-Regulation Shift Theory and Trauma Adaptation

Self-regulation shift theory (SRST) is an extension of SCT (Bandura, 1997) and provides a testable framework related to positive and negative trauma adaptation. SRST draws on a historical tradition in the coping literature that links dynamical systems theory to understand coping adaptation (Neufeld, 1999; Boker, 2001; Levy et al., 2012). According to SRST change across time following trauma may be in a discontinuous manner with threshold shifts (i.e., bifurcations) from one organized state (coherent coping) to another (chaotic coping). Systemic equilibrium is a key driver of behavior and refers to the fact that systems organize around an equilibrium state or steady state. Trauma pushes individuals out of a steady state equilibrium resulting in significant energy (i.e., coping output) being allocated to re-establish a point of equilibrium (i.e., feeling normal again).

The four primary tenets of SRST are: (1) Human beings are self-aware dynamic living systems that require the effective utilization of internal and external resources through feedback mechanisms to self-regulate toward desired goals (Ford, 1987; Bandura, 1997); (2) Under certain conditions, living systems can be pushed into non-linear dynamic shifts from one organized state to another based upon environmental and internal pressures or supports; (3) Coping response output after trauma is comprised of a biopsychosocial action relative to the *perceived* level of disequilibrium (or distance from a state of normalcy or growth) combined with one’s forecast in being able to effectively manage this discrepancy; and (4) A subset of trauma survivors will reach a critical threshold when they believe it is just not possible to regain a sense of control over their recovery (i.e., self-determination violation effect). It is also predicted that survivors can also reach an important threshold where they believe in their ability to recover (i.e., personal agency resurgence).

### Negative Outcomes: A Personal Agency Crisis

The self-determination violation effect has profound implications for perceived autonomy, competence, and connectedness (Ryan and Deci, 2000). Negative consequences include a large increase in negative emotional states (e.g., posttraumatic distress, anger, and despair), impaired coping responses, reduced motivational output, and impaired social interaction. This is a personal agency crisis. SRST is unique, relative to other traumatic stress theories, in that it targets identification of key catalyst variables related to non-linear systemic change across time. Benight et al. (2017) found support for a non-linear negative shift in two samples of motor vehicle accident survivors.

Benight et al. (2017) demonstrated support for the importance of CSE in non-linear shifts in functioning 3 months after a motor vehicle accident. Acute perceived CSE served as a bifurcation factor for the negative non-linear shift in functioning (e.g., posttraumatic symptoms). Importantly, in both samples those who would be considered less affected by the trauma, and therefore less scrutinized for possible psychological challenges, were most at risk for the 3-month negative shift. These results suggest that SRST offers a new lens for understanding critical mechanisms associated with unique trajectories of coping with traumatic stress. The results suggest negative shifts in functioning are interwoven with perceptions of capability to manage the recovery. Positive shifts may also be driven by these same appraisals.

### Positive Adaptation: A Personal Agency Transformation

In contrast to the negative self-determination crisis, a personal agency resurgence is also possible. Effective utilization of internal and external resources (i.e., recovery capital) where systemic equilibrium becomes more attainable allows individuals to regain a sense of mastery and perhaps promote personal transformation. Many trauma survivors explain the experience in some way transformed them into a better person. Instead of reaching the negative threshold where the future is seen as bleak and recovery is perceived as out of reach, positive threshold shifts occur when the goal of future resolution is seen as completely attainable. Cognitions such as “I’ve got this” or “It’s going to be alright” sustain coping efforts and foster hope. We argue that self-perceptions of coping capability (i.e., CSE) will be a pivotal factor in this type of upward shift. Other personal characteristics may also be important to consider (e.g., dispositional optimism, hope, attachment style; Bryant, 2016; Weinberg et al., 2016). Social resources are integral to this dynamic process as social support resources (i.e., enabling hypothesis; Schwarzer and Knoll, 2007) boost individual agency following trauma.

Hobfoll (1989) referred to this dynamic process as a gain cycle where invested resources are rewarded with greater resource accumulation (e.g., self-esteem, social support, life meaning, personal mastery, etc.). These improvements are akin to what Tedeschi and Calhoun (2004) described this as posttraumatic growth and is suggestive of a transformative process. In essence, this process depicts what we often refer to as resilience.

Montpetit et al. (2010) as well as Pincus and Metten (2010) described the importance of a systemic non-linear approach to understand resilience. They described human beings as self-organizing, complex, adaptive systems. Pincus and Metten (2010) highlighted flexibility, openness, and connectivity of the system as key to resilient functioning. When social and individual resources are invested in a flexible open manner within a supportive embedded environment, new systemic configurations become possible.

It should be noted that many theorists have described this personal resurgence (Frankl, 1963; Yalom and Lieberman, 1991; Park et al., 1996; Joseph and Linley, 2005; Joseph and Williams, 2005). By utilizing dynamic non-linear systems analyses, we can observe the underlying systemic shifts or transformations that many trauma survivors describe.

Self-regulation shift theory predicts that some trauma survivors will demonstrate non-linear dynamic state shifts when they reach critical thresholds resulting in a reorganization into a new biopsychosocial state. The person will now experience the world as a different person. Those who are resilient will often refer to themselves as “different,” “deeper,” “more aware,” “more connected,” “stronger” (Tedeschi and Calhoun, 2004). Individuals struggling will describe themselves as “damaged,” “ill,” “broken,” “incompetent,” or “sick.” Shifts between states are possible, yet theoretically, we argue that the system drives toward a new equilibrium or steady state.

Trauma clearly challenges personal agency in ways not ever contemplated by the individual. Systemic adaptation maximally challenges the ability of the self-organizing system to remain flexible, open and connected to ever changing internal and external demands. The system’s ability to reach healthy adaptation equilibrium has critical implications on decision making, affect regulation, and social behaviors. Future research that addresses trauma adaptation with a dynamical systems approach is needed to understand the dynamics of effective and ineffective personal transformation.

## Future Research

Self-regulation shift theory offers some important directions for future studies. Many research questions emerge that await further investigation such as: “Do trauma survivors shift multiple times as they evolve across time?” “Are there pre-existing factors (e.g., trauma history) that predict the timing of a positive or negative shift?” “Do different components of recovery capital (e.g., personal or social resources) play more important roles in shifts at different times and for different groups of individuals?” “Do the number of shifts constitute a more negative or positive trajectory?” These are just a few of the possible questions that need to be addressed. In order to facilitate the needed research in this area, we offer the following information on study design, data collection, and analytic methods. Unique approaches to this line of investigation are necessary in order to more completely capture trauma recovery as a dynamic unfolding process. Luckily, we now have opportunities to harness advanced technology and sophisticated data-analytic methods combined with appropriate longitudinal designs to understand this process in a much deeper way.

## Study Design

The typical longitudinal designs utilized in trauma research are simply not going to provide the level of detail necessary to capture the dynamic adaptation associated with trauma recovery. A Pilots (traumatic stress literature) database search demonstrated that over the past 18 years only 20 studies were published using daily diary assessment information or ecological momentary assessments (EMA). EMA, sometimes called intensive longitudinal data, are taken daily or several times a day and can provide a more fine-grained analysis of individual fluctuations and responses in daily life than typical retrospective assessments (Schwartz and Stone, 1998; Collins, 2006; Shiffman, 2014).

Smartphone technology has catapulted this type of data collection into a whole new world with important implications for behavioral sciences. Smartphones can capture fine grained continuous data related to social interactions, mobility, daily activities, and self-reflections (Harari et al., 2016). Smartphone apps can collect these data utilizing the phone’s accelerometer, GPS, light sensor, Bluetooth connectivity, Wifi, to name a few. Collectively, we are at a revolutionary point in trauma recovery research where we can investigate recovery in a much deeper manner capturing the dynamics of coping adaptation as it unfolds. These methods offer critical information to help test non-linear predictions based on SRST in trauma survivors.

## Data Analytic Approaches for Cusp Catastrophe

To fit data with multiple assessment points to a non-linear model, several non-linear time series analyses are available. We will only mention a few to provide a starting point for future studies. Several analytical approaches are available to test cusp catastrophe models. A cusp catastrophe model is represented by a non-linear shift from one stable state to another. Polynomial regression analysis is an analytical method that is utilized to test cusp catastrophe models (Guastello, 1982, 1987). Similarly, the cusp analysis with a mixed effects model is an extension of polynomial regression analysis and is utilized when the outcome variable is measured more than three times. The lme4 package in R can be used to do this type of analysis (Butner et al., 2014; Bates et al., 2015). The hidden Markov regime switching model (Alpaydin, 2014) can test a non-linear shift from one stable state to another with continuous data points. To run this analysis, R packages such as depmixS4 (Visser and Speekenbrink, 2010) are available. These analytic strategies are only a few of many options (e.g., Grasman et al., 2009; Chow et al., 2015; Chen and Chen, 2017) that can test non-linear shifts in functioning to deepen our understanding of posttraumatic adaptation. These statistical methods provide tools to test theoretical predictions offered by SRST.

## Summary

We have presented a new theory of human adaptation called SRST as a framework for understanding how recovery capital may function as the individual adapts across time. SRST offers testable hypotheses based on dynamical systems theory where a system can shift in a non-linear fashion from one state to another. We outlined in this paper the potential importance of CSE perceptions in the process of personal agency transformation. SRST predicts that when a critical threshold is reached people change in important ways. Those who are gaining strength will often refer to themselves as having become a better person, a deeper individual, or transformed. A negative shift can also occur (Benight et al., 2017). Individuals who are struggling will view themselves as personally damaged. Novel data collection and analytic methods create the catalyst for richer theorizing about how trauma survivors adapt across time. Future research that addresses trauma adaptation from a dynamical systems perspective will help us to see opportunities for unique interventions that promote recovery capital and foster personal agency transformational shifts.

## Author Contributions

CB was involved in drafting the major part of the paper, revising, and agreeing to be accountable for all aspects of the work. AH and KS were involved in drafting some sections, revising, and agreeing to be accountable for all aspects of the work.

## Conflict of Interest Statement

The authors declare that the research was conducted in the absence of any commercial or financial relationships that could be construed as a potential conflict of interest. The reviewer RD and handling Editor declared their shared affiliation.
